# Comprehensive Management of Stroke: From Mechanisms to Therapeutic Approaches

**DOI:** 10.3390/ijms25105252

**Published:** 2024-05-11

**Authors:** Ana Arnalich-Montiel, Alba Burgos-Santamaría, Laia Pazó-Sayós, Begoña Quintana-Villamandos

**Affiliations:** 1 Department of Anaesthesia and Intensive Care, Gregorio Marañón’s University Hospital, 28007 Madrid, Spain; alba.burgos@salud.madrid.org (A.B.-S.); maquin01@ucm.es (B.Q.-V.); 2 Department of Pharmacology, College of Medicine, Complutense University, 28040 Madrid, Spain

**Keywords:** acute ischemic stroke, reperfusion, ischemic cascade, ischemic penumbra, cerebroprotection

## Abstract

Acute ischemic stroke (AIS) is a challenging disease, which needs urgent comprehensive management. Endovascular thrombectomy (EVT), alone or combined with iv thrombolysis, is currently the most effective therapy for patients with acute ischemic stroke (AIS). However, only a limited number of patients are eligible for this time-sensitive treatment. Even though there is still significant room for improvement in the management of this group of patients, up until now there have been no alternative therapies approved for use in clinical practice. However, there is still hope, as clinical research with novel emerging therapies is now generating promising results. These drugs happen to stop or palliate some of the underlying molecular mechanisms involved in cerebral ischemia and secondary brain damage. The aim of this review is to provide a deep understanding of these mechanisms and the pathogenesis of AIS. Later, we will discuss the potential therapies that have already demonstrated, in preclinical or clinical studies, to improve the outcomes of patients with AIS.

## 1. Introduction

Acute ischemic stroke is defined as an abrupt cerebral arterial blood flow cessation, secondary to the occlusion of a blood vessel by thrombi or embolus. This disease represents the second leading cause of death worldwide, behind ischemic heart disease [1], and is the most common cause of disability all over the world [2]. Accordingly, the incidence rate of patients suffering from AIS was 7.63 million in 2019 [3] and continues to increase because of the aging population and the increase in the prevalence of cardiovascular diseases. As a result, AIS has already become an overwhelming major global public health problem. In the light of the above, deep knowledge of AIS and its effective preventive and therapeutical approach is urgently needed. 

The main purpose of this review is to outline the physiopathological mechanisms that develop AIS, focusing on the molecular pathways. In the latter case, we will provide a comprehensive perspective of the potential therapeutic strategies for AIS, including the classical ones (reperfusion therapies and the anesthetic management), the cerebroprotective agents, and, from a future perspective, we will also approach novel emerging strategies that are expected to drastically change the outcomes of stroke patients.

## 2. Physiopathology of Stroke

Stroke is defined as a neurological deficit derived from neuronal cell damage secondary to a vascular dysfunction, which results in an interruption of cerebral blood flow secondary to either a sudden arterial occlusion (“*ischemic stroke*”) or a loss of vascular integrity (“*haemorrhagic stroke*”) [4]. Accordingly, we will focus this review on the acute ischemic stroke (AIS), which represents the vast majority (about 87%) of cases of stroke [5].

Among the potential causes of AIS (detailed in Table 1), embolism is a major one, which can be produced at the expense of a vessel´s atherosclerotic plaques or due to cardiac thrombi (normally associated to atrial fibrillation). In situ thrombosis of cerebral arteries is another important cause of stroke. Furthermore, cervical artery dissection and arterial inflammation can also hasten this disease. Regarding potential vessels involved in AIS, the middle cerebral artery (MCA) is the most common one, representing 70% of all the cases [6].

Immediately after cerebral blood flow is interrupted, cerebral ischemia is delayed and partially compensated by collateral blood circulation through leptomeningeal anastomoses or the circle of Willis.

Thus, the affected cerebral tissue will be promptly divided into two well distinguished areas. On one hand, the ischemic core or necrotic area will appear distant from the collateral blood vessels, associated with total or insufficient cerebral blood flow (CBF) to satisfy the metabolic requirements of the brain. This ischemic core is responsible permanent cerebral damage. On the other hand, the “*penumbra area*” will also show up, defined as the surrounding area of the ischemic core where blood perfusion is visibly reduced but still enough to ensure the neurons’ survival. If the penumbra area is not finally salvaged because CBF is not restored in time, it will progressively be recruited to the infarct core [7].

**Figure 1:**
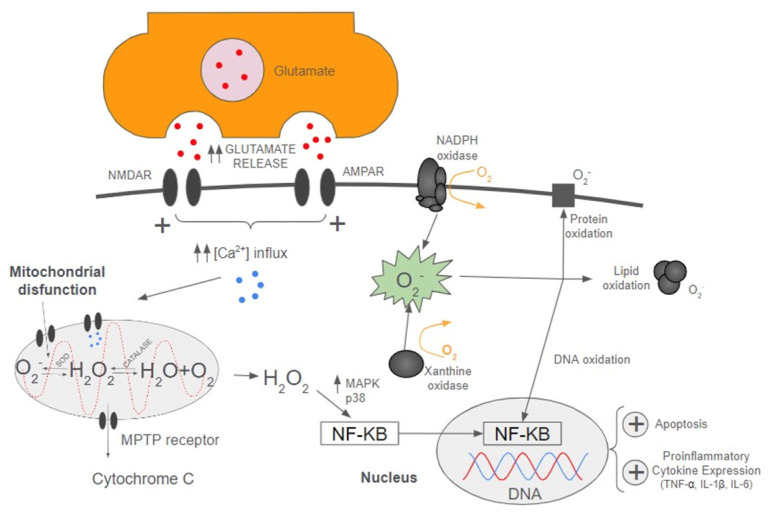
Interaction between neuroinflammation, oxidative stress and apoptosis in AIS.

The problem worsens once cerebral perfusion is reestablished, bringing oxygenated blood back into ischemic brain tissue, generating further damage in the penumbra area and amplification of the “ischemic cascade”, producing the “*ischemia-reperfusion injury*” phenomenon. Deep understanding of the underlying mechanisms of the ischemic cascade is essential to improve the outcomes of stroke patients, as it will provide a wide margin in the search for therapies addressed towards these mechanisms that we will discuss later. Below, we will investigate these mechanisms (some of them are detailed in Figure 1). 

### 2.1. Disruption of Brain Energy Metabolism

The absence or decrease of cerebral flow destabilizes the relation between the brain´s energy input and its demand. As neurons from the penumbra area receive between 20–40% less blood flow than normal, they become electrically silent as their metabolism is considerably reduced. On the other hand, glucose and oxygen deprivation within the ischemic core results in a severe decrease in ATP production within the first 5 min of artery occlusion, causing irreversible brain damage [8].

Glucose normally enters neurons through GLUT receptors, and then it is intracellularly transformed to glucose 6-phosphate, which is metabolized through glycolysis process into to molecules of pyruvate. In the presence of oxygen, pyruvate enters mitochondria and produces ATP through oxidative phosphorylation. However, under hypoxic circumstances, pyruvate can be also reduced to lactate, generating a significantly higher amount of ATP (10-fold or higher), which is transported extracellularly by monocarboxylate transporters (MCTs). 

Glycolysis and oxidative phosphorylation are the two preferred ways of ATP production in neurons and astrocytes, respectively. Astrocytes are the most prevalent glial cells in the brain and play a crucial role during cerebral ischemia, providing glucose and lactate to the extracellular space so that the impaired neurones can use it, preventing them from death. In fact, astrocytes are the only cells within the brain capable of storing glucose through gluconeogenesis and also have the highest glycolytic activity. This is clinically relevant, as noted in many studies, where lactate has been shown to have a neuroprotective effect by reducing stroke progression [9].

### 2.2. Glutamate-Mediated Excitotoxicity

Cerebral ischemia leads to a derangement in the ionic gradient, which happens because of the disruption of ATP-dependent transporters, such as Na^+^/K^+^ ATPase, Ca^2+^ ATPase, and Na^+^/Ca^2+^ channels within the neuronal cells. All this results in an overload of free radicals and the accumulation of glutamate in the neuronal extracellular space. 

Glutamate is an excitatory amino acid that acts by overstimulating N-methyl-D-Aspartate (NMDA) and α-amino-3-hydroxy-5-methyl-4-isoxazolepropionic acid (AMPA) receptors, leading to a massive cellular influx of calcium ions into the neuron. AMPA activation causes disbalance in the osmotic cell gradient by increasing sodium and chloride influx, which accentuates neuronal swelling and cell lysis [10]. 

Activated astrocyte is essential for neuroprotection in ischemia, as it participates in glutamate uptake and counters the increase of extracellular levels of this neurotransmitter [11]. 

### 2.3. Neuroinflammation and Blood to Barrier Disruption

After ischemic stroke, there is an excessive inflammatory response, which leads to progression of the primary ischemic brain insult, blood-brain-barrier (BBB) disruption, and brain oedema. Cellular debris and associated protein fragments called damage associated molecular proteins (*DAMPs*) participate in the early activation of the innate system response through its union to TRL2 and TRL4, which are patter recognition receptors that are present in the circulating immune cells, such as neutrophils, but also resident CNS cells, such as microglia cells and astrocytes. 

Microglia cells are non-activated macrophages within the brain that play a decisive role in stroke neuroinflammation. It can be transformed into either a proinflammatory/proapoptotic phenotype (M1) or an anti-inflammatory/neuroprotective phenotype (M2). M1- induced pro-inflammatory responses contribute to accentuating the brain injury, with BBB loss of integrity and further migration of inflammatory cells to the impaired tissue. This response is associated with the release of several cytokines, such as TNF-α, IL-1β, and IL-6, as well as proapoptotic substances, such as TNF-related apoptosis-induced ligand receptor (*TRAIL-R*) and Fas Ligand (*FaSL*). 

In contrast, M2 activity has a neuroprotective effect, the main role of which is becoming phagocytes of apoptotic cells and cellular debris; in the meantime, they produce several growth factors and neurotrophic proteins, such as the brain-derived neurotrophic factor (BDNF) and the insulin-like growth factor 1 (IGF-1) [12]. 

**Figure 2:**
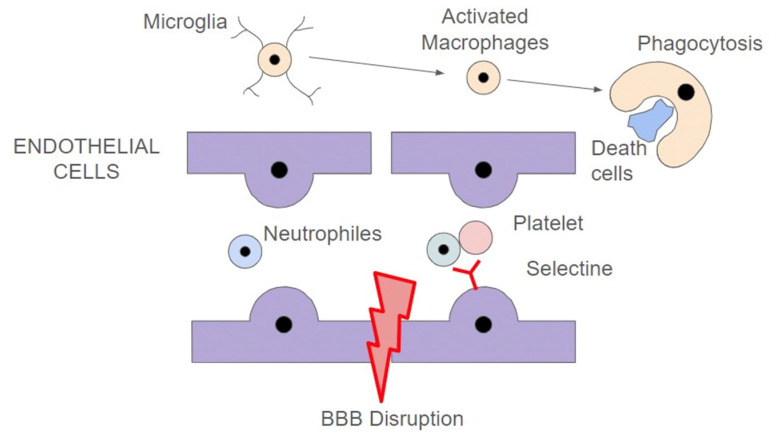
Blood-brain-barrier (BBB) disruption, clot formation, and microglial activation as some of the underlying mechanisms of neuroinflammation in AIS.

Furthermore, microglial cells participate in the upregulated expression of adhesion molecules within the damaged endothelium, such as intercellular adhesive molecule type 1 (ICAM-1), P-selectine, and E-selectin, as well as cytokine release triggering the recruitment of other macrophages and circulating immune cells, such as dendritic cells, lymphocytes, astrocytes, and neutrophiles (detailed in Figure 2) [13]. 

Activated neutrophils release substances that destroy extracellular matrix and tight junctions, such as elastase, matrix metalloproteases (MMP), inflammatory IL-1β, and reactive oxygen species (ROS). This results in a cerebral BBB breakdown and, consequently, it produces cerebral oedema. This latter is due to the entry of H_2_O and plasmatic proteins into the cerebral interstitial space through the fenestrated damaged capillaries. The extent of BBB disruption and the delay in blood reperfusion is directly associated with a higher risk of haemorrhagic stroke conversion [14]. In addition, astrocytes are the CNS cells that suffer from swelling to a greater extent.

In parallel, activation of the innate response triggers the adaptative immune response (T and B cells) and the complement system. In this sense, astrocytes play, together with microglial cells, a very important role on neuroinflammation after stroke. They can modulate the inflammatory response by inhibiting T cells and, in addition, they activate component C3 and C5, which are involved in the phagocytosis processes, removing debris and dead cells from the infarct area.

Looking beyond cerebral tissue, it has been well documented that, following AIS, there is an interaction between the brain and peripheral immune cells, named “*brain-spleen inflammatory coupling*”. In response to cerebral ischemia, an upregulation of the sympathetic tone occurs and enables the release of pro-inflammatory cells, which migrate from the spleen into the brain, contributing to the exacerbation of the inflammatory response. In contrast, activation of the splenic parasympathetic tone induces TNF-α activity suppression, being associated with a neuroprotective effect and a reduction in the size of the infarcted area [15]. 

Once reperfusion has been established within the damaged tissue, adhering leukocytes are rapidly bound to activated platelets constituting a “clot” and predisposing to microvessel occlusion and neurological deficit, despite successful reperfusion therapy. This is known as the “*non-reflow*” phenomenon [14]. Thrombin presence in the clot triggers the expression of more endothelial adhesive proteins and a further leukocyte’s vessel adhesion. 

### 2.4. Increase in Oxidative Stress Status

Once cerebral perfusion is disrupted, lack of glucose and oxygen bioavailability decelerates mitochondrial oxidative phosphorylation, critically compromising ATP synthesis. Consequently, mitochondrial electron transport chain, redox active enzymes, and reducing equivalent carriers (NADPH, succinate, ubiquinol, free fatty acids, acyl-CoA) are maximally reduced. 

Respiratory complex I is one of the main sites of ROS generation in the mitochondria. During reperfusion, ischemic neurons suffer an important increase in ROS production through the mechanism of Reverse Electron Transport (RET). Under hypoxic circumstances, reverse succinate dehydrogenase activity is enhanced, leading to an overproduction of succinate and, consequently, an increased production of electrons. RET is produced when these electrons are transferred along the Respiratory complex I from ubiquinone to NADH dehydrogenase, reducing NAD+ to NADH [16]. 

Transmembrane protein NAPDH oxidase (NOX) uses molecular oxygen to produce one of the major sources of ROS, the superoxide anion. This complex enzyme is composed of seven subunits (NOX-1, NOX-2, NOX-3, NOX-4, NOX-5, Oxidase 1 and Oxidase 2) and its expression has notably been increased in experimental models of stroke [17]. 

Furthermore, derangement of the ionic membrane gradient is followed by intracellular influx of calcium ions (Ca^2+^), leading to mitochondrial permeability transition pore (MPTP) opening and cytochrome C release to the cytoplasm, which activates signalling pathways of apoptosis. Additionally, the mitochondria releases Ca^2+^ into the cytoplasm, contributing to a further increase of Ca^2+^ intracellular levels [2].

In summary, mitochondrial disfunction comes secondary to many cellular derangements (ATP depletion, RET, calcium overload, and changes in pH). This contributes overproduction of reactive oxygen species (ROS), leading to a severe neuronal impairment and, ultimately, to cell death processes. 

Apart from mitochondria, there are two other main sources of ROS production, which are also triggered in AIS. On one hand, xanthine oxidase (XO) is an enzyme that has been noted to be overexpressed in AIS and that catalyzes oxidation of hypoxanthine to xanthine; later, it transforms xanthine into urat, producing O2 and ROS [18]. 

On the other hand, cyclooxygenase, cytochrome P450 enzyme, or lipoxygenase represent other less common pathways of ROS production.

ROS species, such as superoxide and peroxynitrate, are involved in the DNA transcription processes during cerebral ischemia, promoting an enhanced inflammatory response, cell injury, and apoptosis. Some of the nuclear transcription factors triggered by ROS in AIS are nuclear factor kappa B (NF-κB.), interferon regulatory factor 1 (IRF1), hypoxia-inducible factor 1 (HIF-1), activator protein 1 (AP-1), p53, and STAT3. 

To counter the ROS production, astrocytes produce glutathione (GSH), a potent antioxidant which is mainly produced by these cells within the brain [11]. 

### 2.5. Apoptosis

“Apoptosis” is a strictly controlled process that aims to destroy harmful cells in order to maintain cellular homeostasis [19]. It is triggered by either intrinsic or extrinsic pathways. The intrinsic pathway consists of the previously mentioned glutamate-mediated excitotoxicity process, followed by a calpain-mediated cytotoxic response in the nucleus and cytoplasm. Additionally, ROS production causes oxidative damage to lipids, proteins, and DNA, triggering apoptosis through many different pathways. First, it triggers p53 upregulation, which promotes transcription of pro-apoptotic genes (Noxa, Bcl-2), and stimulates pro-apoptotic proteins (Fas, DR-x) [20]. Second, there is an overexpression of mitogen-activated protein kinase (MAPK) and nuclear factor NF-κB [21]. Furthermore, apoptosis also occurs via downregulation of PI3K/Akt signalling [22]. Finally, release of H_2_O_2_ species deranges mitochondrial membrane permeability, promotes chromatin condensation, and stimulates endoplasmic reticulum stress. 

Cerebral ischemic damage also activates the extrinsic pathway, which can be activated independently or simultaneously to the intrinsic pathway mechanisms. It can be mediated through the activation of neuronal membrane receptors, such as TRAIL-R and FasL, leading to an overexpression of caspase-8 and caspase-3, thereby activating apoptotic signalling [23] 

### 2.6. Other Mechanisms of Regulated Cell Death

Oxidative stress and increased intracellular calcium levels induce permanent neuronal damage, which induces expression of phosphatidylserine (PS) on the membrane cell. This expression represents an “eat-me” signal, which enables recognition and phagocytosis by microglia cells and activation of complement pathway through C1q and C3.

Apart from phagocytosis, AIS is related to mechanisms other non-caspase-mediated cell death mechanisms, such as ferroptosis, parthanatos, and pyroptosis [22]. Ferroptosis consists in abnormal metabolism of iron and causing lipid peroxidation, resulting in membrane rupture. In parthanatos, cell death is mediated by overexpression of a nuclear chromatin-associated enzyme named poly ADP-ribose polymerase 1 (PARP1), which plays a major role in recognizing and repairing DNA breaks through NAD+ and APT consumption. The previously described energy failure of AIS triggers PARP overactivation, resulting in DNA fragmentation and chromatin condensation, swelling, and membrane cell rupture. Finally, pyroptosis is a programmed type of cell death mediated by gasdermin D. This protein forms membrane pores and is the binding site for caspase-1 and caspase-11. This junction triggers the release of pro-inflammatory cytokines, like IL-1β and IL-18, promoting cellular destruction.

## 3. Influence of Comorbidities in the Pathogenesis of Stroke

Smoking and diabetes are frequent and modifiable risk factors of AIS. Furthermore, both diseases have been shown to worsen the outcomes after cerebral ischemia, as they accentuate the ischemic cascade mechanisms of deranged energy metabolism and mitochondrial disfunction that results in higher levels of ROS production. Additionally, they both alter BBB function and cerebral blood flow, leading to a more extensive cerebral oedema. Furthermore, nicotine exposition decreases neuronal glucose uptake by decreasing the GLUT 1 and GLUT 3 expression in these cells [24]. Finally, it is also remarkable that hyperglycaemia is related to a downregulation of antioxidants, such as SOD, catalase, and glutathione peroxidase [25].

## 4. Endogenous Mechanisms for Countering Cerebral Ischemia

### 4.1. Mitochondrial Quality Control (MQC)

As we have previously discussed, mitochondria play an essential role in the pathogenesis of AIS. In this sense, MQC mechanisms enhance neuronal survival by reversing mitochondrial damage and energy failure. It also palliates oxidative stress and reduces ROS-mediated cellular damage. Among all the MCQ pathways, mitochondrial biogenesis, mitochondrial fusion, mitochondrial fission, and mitophagy seem to be the best-known ones. Thus, they are potential novel therapeutic targets for AIS, as we will detail below.

Mitochondrial biogenesis is a process based on mitochondrial growth and DNA replication and division in order to generate new organelles. It is regulated by several signalling pathways, like SIRT1-PGC-1 α and AMPK-PGC-1α [26]. SIRT1, AMPK, and mTOR are proteins that are overexpressed in stroke, activating proliferator-initiated receptor gamma and coactivator 1alpha (PGC-1 α), which is a nuclear transcriptional co-activator of several transcription factors involved in most of the mitochondrial functions (biogenesis, ROS production, and mitophagy). 

Furthermore, mitochondrial fission enables damaged mitochondria to restore themselves by removing and replacing damaged mitochondrial DNA, proteins, and lipids with new components. OPA 1 is a key regulator gene involved in this process. 

Finally, mitophagy is responsible for removing selectively dysfunctional mitochondria and requires the activation of specific pathways, PTEN-induced kinase 1 (PINK1) and Parkin being two of the most studied ones [27]. In this sense, astrocytes play an important role, receiving damaged mitochondria and transferring healthy mitochondrial to neurones [11].

### 4.2. Angiogenesis

Angiogenesis is defined as a coordinated process of remodelling and growth of new blood vessels within the penumbra area to respond to the ischemic insult, being a key mechanism to enhance the neuron´s survival and reduce cerebral damage. Astrocytes participate actively in this process, modulating cerebral blood flow and vascular remodelling [11]. Hypoxia inducible factor (HIF) is a transcriptional factor that is overexpressed in hypoxic conditions and promotes angiogenesis through VEGF release. 

## 5. Potential Therapies in AIS

At present, reperfusion therapy, either by intravenous thrombolysis (IVT), endovascular thrombectomy (EVT), or a combination of both, is considered to be the most effective treatment for cerebral ischemia. 

However, these therapies can only be administered within a short interval of time and are not indicated for all AIS patients, leaving significant room for improvement in the management of this group of patients.

As previously mentioned, as new mechanisms for ischemic brain damage are being discovered, the spectrum of therapeutic possibilities is progressively widening. Specifically, cerebroprotective therapy is gradually becoming more frequently used as a coadjuvant therapy in AIS, spreading out promising results which point to a significant improvement in the stroke patient´s outcome. Furthermore, other emerging therapies, such as cellular therapies, remote ischemic conditioning, or mitochondrial quality control (MQC) target agents, are gaining strength as an attractive alternative to the classical treatments. 

We will now approach all the potential therapies for AIS, pointing out which ones have demonstrated beneficial effects in the clinical and preclinical scope.

### 5.1. Reperfusion Therapy

#### 5.1.1. Endovascular Thrombectomy (EVT)

EVT consists in a mechanical withdrawal of the blood clots that are obstructing an artery blood flow, either intracranial or a carotid vessel. This procedure has demonstrated a dramatic improvement in the outcomes of patients with AIS due to large vessel occlusion (LVO). 

As neuroimaging techniques have developed, EVT is indicated in AIS patients who have suffered a large arterial occlusion along the anterior cerebral circulation within the first 24 h (preferably in the first 16 h) from the onset of symptoms and that fulfill the appropriate criteria (NIHSS scale ≥ 6, ASPECTS score ≥ 6) [28]. 

AIS treatment changed quite notoriously in 2018 after dissemination of the results of two randomised clinical trials: DAWN and DEFFUSE-3 [29,30]. Both studies validated the penumbra area as a good indicator for patients who would respond favourably to reperfusion, and both expanded the therapeutic window for EVT from 6 h up to 24 h. They demonstrated that thrombectomy in AIS patients and MRI/CT imaging criteria of salvageable ischemic penumbra could undergo late time reperfusion (within the first 24 or 16 h, respectively, from the onset of symptoms) and still maintain significantly better functional outcomes than patients treated with medical therapy. This benefit was demonstrated as a reduction in neurological disability and a better modified Rankin scale 3 months after the intervention. 

#### 5.1.2. IV Thrombolysis

Intravenous thrombolysis (IVT) with a tissue-type plasminogen activator (tPA) named alteplase has been the only and preferred treatment for 25 years; it is currently the only FDA and EU approved medical treatment for patients with acute ischemic stroke (AIS) [23]. It is a second-generation thrombolytic agent that acts by converting plasminogen to the proteolytic enzyme plasmin, which breaks down fibrin and fibrinogen dissolving cerebral blood clots. As opposed to EVT, it is indicated in all types of ischemic stroke, no matter where the occlusion is placed. However, the therapeutic time window in IVT is narrower than in EVT, and it cannot exceed the first 4.5 h [31,32]. Apart from its early administration, it requires discarding intracranial haemorrhage in the cranial CT, which is an absolute contraindication for this therapy. It has been demonstrated that earlier IVT treatment is associated with better outcomes and lower risk of developing secondary intracranial haemorrhage, which represent its most alarming complication.

In some cases, coadministration of endovascular thrombolysis near the cerebral blood clot can be given to further improve the outcomes of patients undergoing EVT with large vessel occlusion, reducing the risk of non-reflow phenomenon or distal occlusion following this procedure [33,34].

#### 5.1.3. Heparin, Antithrombotic and Antiplatelet Agents:

Intravenous (IV) heparin is always administered during EVT to minimize catheter-induced embolic and thrombotic events. Heparin activity should be monitored by measuring activated clotting time (ACT) every 30 to 45 min, aiming to stick to the range in between 250 to 300 s [35]. 

Based on thereon, heparin administration can be titrated and fully reversed with protamine once the EVT is concluded. However, this is generally not necessary, except for complicated patients with high risk of intracranial bleeding (due to arterial perforation or dissection). 

On the other hand, in cases of failing revascularization or embolism complications, additional therapy must be considered. Postprocedural infusion of antithrombotic agents such as Argatroban, platelet glycoprotein IIa/IIIb receptor antagonists like Eptifibatide and Tirofiban, or antiplatelet agents like Glenzocimab have been shown to improve reperfusion effectiveness and durability. Therefore, they may be considered as a coadjuvant therapy associated to IVT in cases of high risk of re-occlusion [36,37].

Antiplatelet therapy has been widely used and plays a crucial role in management and secondary prevention of AIS. This group of drugs inhibit platelet aggregation and blood clot formation through different mechanisms. Aspiring acts produce an irreversible inhibition of platelet cyclooxygenase (COX) enzyme, interfering in the transformation of arachidonic acid into thromboxane A2, thus balking platelet aggregation. On the other hand, clopidogrel is a thienopyridine that irreversibly blocks P2Y12 adenosine diphosphate (ADP) receptor. Finally, ticagrelor acts in the same target as clopidogrel, but it has a reversible action [38].

Unlike what it is recommended in patients with acute coronary syndrome, in AIS there is no benefit in using combination of antiplatelets (what is called “dual antiplatelet therapy”) during the acute phase of the disease, as it can increase the risk of bleeding complications without a significant decrease in the risk of stroke (MATCH trial and CHARISMA trials) [39,40].

#### 5.1.4. Anesthesia for EVT

Patients with AIS can undergo EVT under General Anesthesia (GA) or “*Monitored Anesthesia Care*” (MAC). This last choice includes a broad range of alternative anesthetic techniques, ranging from local anesthesia up to deep sedation. Eventually, anesthetic approaches can be interchangeable, and a quick conversion from MAC to GA may be required to secure the airway and stabilize the patient. This situation may be triggered by the emergence of an acute adverse event (hypoxemia or bronco-aspiration) or the lack of cooperation from the sedated patient.

For many decades, there has been controversy about which anesthetic technique will provide better outcomes in AIS patients. There is no scientific consensus on which of these two techniques involves a better outcome, as detailed below:

- Most of the evidence supporting superiority of GA versus MAC is based on three single center randomized controlled studies (RCT) [41,42,43]. It is important to consider that these studies were conducted with no more than one hundred patients, and therefore it is not possible to extrapolate results to general population.
- There are, somehow, multicentric RCT studies like AnnStroke [44] and the GASS [45] and the AMETIS trials [46], which have not demonstrated differences in clinical outcomes between GA and MAC. Furthermore, two recent meta-analyses [47,48] have ratified that there were no difference in functional recovery, 3-month mortality or major complications between groups despite GA providing a better recanalization rate after EVT versus MAC.
- In contrast, there are some other studies that showed worse functional outcomes and higher mortality in patients who received GA compared those who received MAC [49,50,51,52,53].

The disparity of the results may be a result of some methodological limitations of the studies described above. First, there is a potential selection bias, as the choice of the anesthetic approach may be influenced directly by the severity of the stroke. In this sense, patients with higher scores on the National Institutes of Health Stroke Scale (NIHSS) at admission will have a higher probability of receiving GA. Second, most of the studies did not include the impact of the anesthetic strategy on the outcome as the main goal of the study. Another issue is that the data of anesthetic management and hemodynamic variables were not well reported, and their differences may influence the outcomes. Additionally, sometimes the anesthetic technique was performed by an anesthetic clinician or a non-anesthetic clinician instinctively, generating differences in the management and outcome between both groups. Finally, many studies have typically defined GA as the presence of an endovascular tube regardless of the drugs administered, depth, or sedation. 

In the light of the above, we cannot consider that there is evidence enough to support the superiority of one anesthetic technique above another and we need to individualize the decision in each case based on patient and procedural factors, as well as the resource availability and the experience of the center. In this sense, it is very reasonable to believe that an adequate management of hemodynamics as well as other physiological parameters (blood glucose, 0_2_, C0^2^, body temperature) may be a better guarantee of neuroprotective strategy and good postprocedural outcomes than the choice of the anesthetic technique itself [54].

### 5.2. Cerebroprotective Therapy

Over the last four decades, the pathogenic mechanisms of AIS were clarified, and several trials have been conducted using novel therapies that aimed to enhance brain tissue tolerance to ischaemia and to avoid secondary injury to the ischemic tissue. Previously called “neuroprotectants”, they were subsequently renamed as “cerebroprotectants” once they were known to be involved in protection of non-neuronal CNS cells such as endothelium, astrocytes, and microglia. These drugs target several components of the ischaemic cascade (interfering in the physiopathological mechanisms that were detailed above); some of them are multitargeted. They are intended to be used in combination with recanalizing therapy (EVT or IV thrombolysis) to improve the clinical outcomes in patients with AIS. 

Cerebroprotective therapy has been widely tested, showing promising results in clinical and preclinical trials. However, it has not been implemented in the clinical practice. We will now describe different types of cerebroprotectants that have been demonstrated to have beneficial effects for combating cerebral ischemia. 

#### 5.2.1. Nerinetide

NA-1 (nerinetide) is a 20 amino acid peptide that prevents the postsynaptic density protein 95 (PDS-95) from binding to NMDA receptor (NMDAR). As a result, it downstreams glutamate excitotoxicity mechanisms (such as excessive Ca^2+^ influx and nitric oxide generation) described above [55]. After many years of disappointment with neuroprotectant research in humans, the ESCAPE-NA1 trial was one of the first studies showing encouraging results, demonstrating that NA-1 can enhance the outcomes of patients with AIS undergoing EVT who did not receive concurrent iv alteplase [56]. Previously, preclinical evidence in models of stroke had already demonstrated a significant reduction in the infarct size and improvement of neurological outcomes in the NA-1 group [57]. To investigate the presumable interaction between Nerinetide and tPA, a new phase III clinical trial with patients who have not received iv tPA is now being performed [*ESCAPE-NEXT*; *NCT04462536*]. Additionally, another trial that aims to investigate the outcomes of AIS patients receiving Nerinetide in the prehospital field is also ongoing [*NCT02315443*]. 

#### 5.2.2. Sovateltide (IRL-1620)

Sovateltide is an endothelin B receptor (ETBR) selective agonist and synthetic analogue of endothelin 1, which has been associated with beneficial effects in model of stroke in rats [58]. It acts by promoting mitochondrial biogenesis and has anti-apoptotic effects through the regulation of PGC-1 α expression. These results have been extrapolated to humans in a phase III trial with 40 patients who received Sovateltide within the first 24 h after stroke onset. Therapy with Sovateltide significantly improved their neurological outcomes 90 days post-treatment [59]. 

#### 5.2.3. Activated Protein C (APC) 

APC is an activated plasma serine protease that constitutes a multitarget agent for the treatment of stroke. It has very well-known anticoagulant, antiapoptotic, and anti-inflammatory properties. Furthermore, APC has a neuroprotective action as an agonist of protease-activated receptors 1 and 3 (PAR-1, PAR-3), which are G protein-coupled receptors that activate anti-inflammatory signalling in neurons and endothelium. 3K3A-APC is a recombinant variant of APC, which has been designed for stroke models as it has a highly reduced anticoagulant activity. In a murine model of stroke, early administration of 3K3A-APC has been demonstrated to have neuroprotective effects in AIS [60]. These results have been also ratified in a phase II clinical trial, where APC showed a trend towards lower haemorrhage rates in patients treated in combination with reperfusion therapy [61]. Although mechanisms underlying this effect have not been clarified yet, APC seems to protect the brain from tPA´s toxicity in AIS, therefore being part of the group of novel drugs called “tPA helpers”. 

#### 5.2.4. Human Urinary Kallidinogenase (HUK) 

HUK is a glycoprotein extracted from male human urine that enhances cerebral perfusion within the penumbra area through two different pathways. First, HUK activates the kallikrein–kinin system (KKS), promoting angiogenesis and neuroprotection. In addition, HUK catharizes hydrolysis of low molecular weight kininogens to vasoactive kinins, the latter being responsible for cerebral vasodilatation. 

For more than a decade, HUK has been approved by the Chinese Food and Drug Administration and used in stroke patients. Several studies have demonstrated the beneficial effects of HUK in the treatment of patients with AIS by improving the neurological outcomes of the patients [62].

#### 5.2.5. Edaravone (MCI-186) 

Edaravone is another selective cerebral vasodilator and free radical scavenger that reduces oxidative stress and lipid peroxidation in AIS patients. This cerebroprotectant agent has already been approved for clinical use in AIS in countries like China and Japon (since 2001). Since its beneficial effects were demonstrated in preclinical studies [63], several clinical trials have been performed in humans to corroborate these results, mostly in Asian populations. A meta-analysis was carried out including 19 clinical trials and showed that patients treated with Edaravone had better neurological outcomes and lower rates of mortality than patients treated with a placebo [64]. Furthermore, it has been demonstrated that a combination of Edaravone and tPA maintains a neuroprotective effect and, at the same time, it attenuates tPA risk of haemorrhagic transformation (thus, it is considered another tPA helper) [65]. This might be explained by the fact that Edaravone inhibits the expression of the matrix metalloproteinase-9 (MMP-9), encouraging BBB integrity within the damaged ischemic tissue [66].

#### 5.2.6. ApTOLL

TLR4 is a protein involved in the recognition of pathogen-associated molecular features that triggers both innate and adaptative immune responses; it also induces the inflammatory response [67]. Results from preclinical studies performed in a model of stroke in rodents [68] have shown that a TLR4 antagonist, known as ApTOLL, can reduce the infarct volume and enhance their neurological outcomes. Recently, a clinical phase IB/IIA trial has demonstrated that the administration of TLR4 antagonist is safe and maintains its neuroprotective effects when reproduced in AIS patients undergoing EVT [69].

#### 5.2.7. Fingolimod (FTY720)

FTY720 (Fingolimod) is a sphingosine-1-phosphate (S1P) receptor modulator that was approved by the Food and Drug Administration (FDA) after demonstrating its beneficial effects in multiple sclerosis by reducing disability progression [70]. This drug prevents lymphocytes from exiting the lymph node into the vascular territory, thus promoting downregulation of the inflammatory response and brain damage after stroke. FTY720 seems to have several other neuroprotective effects, such as BBB integrity maintenance or apoptosis regulation. Several clinical trials have demonstrated that Fingolimod administered within the first few days after stroke onset, in combination or not with tPA alteplase, has a beneficial impact on the outcomes of AIS patients [71,72]. 

### 5.3. Cellular Therapies

These promising regenerative strategies are focused on stem cell transplantation. It enables replacement of damaged tissue cells and secretion of mediators that can palliate the main mechanisms of the ischemic cascade (neuroinflammation, oxidative stress, apoptosis). Stem cell engraftment is not essential to repair brain infarcts; this can also occur through stem-cell secretomes, which can release growing factors, cytokines, micro RNA, and other mediators to promote neurogenesis [73,74]. 

Depending on the origin of the stem cells, they may vary in terms of proliferation, migration, and differentiation features. Stem cell therapy (SCT) from adipose tissue, embryo, bone marrow, umbilical, and neural origin has already been tested in experimental models of AIS [75,76,77,78]. Though it is still too early to confirm that these therapies are secure and well-tolerated in humans, it seems that these therapies will dramatically enhance the treatment and the outcomes of patients with AIS. 

Astrocyte co-transplantation with neural stem cells (NSCs) has already proven to be a good strategy in a murine model to increase survival and neuronal differentiation of NSCs following ischaemic stroke in rats compared with NSC transplantation alone [79].

### 5.4. Remote Ischemic Conditioning (RIC)

Remote ischemic conditioning (RIC) is a non-invasive procedure in which exposure of stroke patients to short intermittent intervals of ischemia and reperfusion on the upper limb can activate endogenous mechanisms of neuroprotection against hypoxia and promote neuronal repair. These mechanisms have already been demonstrated in preclinical models and are related to processes of neurogenesis, angiogenesis, axon regeneration, synaptogenesis, and remyelination [80,81]. 

A multicentric clinical trial performed in China with 1893 patients with AIS demonstrated an outstanding neuroprotective effect by reducing the infarct volume and brain oedema and improving neurological post-stroke function [82]. In contrast, another multicentric trial that enrolled stroke patients who initiated RIC in the prehospital setting and continued during their hospital admission showed no significant improvement in functional outcome at 90 days [83]. This latter study has two main limitations related to the study sample: first, it also included haemorrhagic stroke patients and second, its size was considerably reduced due to higher rates of patients with transient ischemic attack than expected.

### 5.5. Inhibition of Reverse Electron Transport (RET)

It has been hypothesized that RET inhibitors may produce neuroprotection by interfering with the pathogenesis of stroke. Among this group of drugs, we must point out the efficacy of metformin [84]. This drug has been demonstrated to block ischemia-reperfusion induced oxidative stress and palliate ROS production stroke damage in animal models.

## 6. Discussion and Future Perspectives

As we gain a deeper knowledge of the pathogenesis of stroke, a wide range of novel emergent therapies are bursting into the AIS management, developing promising results towards cerebral penumbra area recovery and the patient´s clinical outcomes. As we have previously seen, most of these therapies are focused on the preclinical field and they still need to be ratified in humans.

Translating these positive results to the clinical practice is an arduous and challenging task for many different reasons. First, because differences in dosages and timing of administration between humans and animals do exist and are not easy to manage, they confer a negative impact on the results. Second, current animal models of stroke might be inadequate in resembling the complexity of the pathogenesis previously described for humans with AIS; thus, it might be the time to produce novel ones. Finally, all the preclinical studies use the infarct volume area as the primary endpoint as it is easier to measure. To enhance the power to detect meaningful cerebral damage, it might be necessary to also include behavioural endpoints that resemble the modified Rankin Scale, which is a widely used clinical score used to measure global disability in humans with AIS [85].

On the other hand, deeper research into the underlying mechanisms of AIS is necessary to search for effective and novel therapies that could produce a clinical impact in AIS. As we already know, progression of this disease is related to the activation of different pathways that act in coordination with and interact with each other. In this sense, the concept of the neurovascular unit (NVU) is gaining strength as it refers to the narrow and coordinated communication between different cells (glia and neurones) with the surrounding endothelium to maintain cerebral perfusion and homeostasis [86]. As neurons can only survive within NVU, searching for pleiotropic agents, which will address to different targets and cells within NVU, will exponentially boost the beneficial effects in AIS patients compared to single-target drugs.

Stroke has become a concerning major global public health problem, and it therefore requires an urgent solution. We have commented on novel drugs, some of them pleiotropic, which have already given hope for the better management AIS in preclinical trials. However, these drugs have a significant handicap: in order to ratify their benefits in AIS and to approve them in the clinical field, a significant investment of time and resources are required. Both conditions are limited and complicated to achieve. Thus, it is time to reconsider classical drugs that were approved for treating different diseases but somehow, they are involved in similar pathophysiological mechanisms than the ones of AIS. In this sense, cyclosporine A (used to treat post-transplant immunosuppression), Ca^2+^ channel blockers like nimodipine (used to treat aneurysmal subarachnoid haemorrhage), or antidiabetics (metformin) have already demonstrated to provide neuroprotective effects in preclinical [87,88] and clinical trials [89], although they still have not shown a clear benefit in models of AIS. However, further robust and well-designed trials need to be carried out before confirming this hypothesis. Another possibility is to make some improvements in the structure of neuroprotective drugs to obtain a better BBB passage, better cerebral bioavailability, a greater potency, and a reduction in the side effects. 

Neuroinflammatory cascade was found to be activated within 36 to 133 min after stroke and progresses rapidly [90]. We do know now that interfering as soon as possible with the mechanisms of the ischemic cascade is crucial, as it will enable recovery of the penumbra area before it is too late and permanent cerebral damage occurs. To respond to this demand, it would be desirable to elaborate algorithms for prehospital diagnosis and management through mobile stroke units, to ensure early administration of cerebroprotective therapy to the stroke patient prior to hospital admission, and to encourage team training and cooperation among health workers.

From a future perspective, it is necessary to highlight that great expectations are set by the emerging nanomedicines (which include micelles, nanoparticles, dendrimer, and exosomes) and their ability to deliver fast and selectively-specific drugs to controlled sites within the infarcted brain. These therapies can either reach the thrombus site (to provide thrombolytics enhancing its effectiveness and security) or alternatively the NVU (bringing cerebroprotection to the injured neural and glial cells). 

## 7. Conclusions

Aging population and increasing prevalence of cardiovascular risk factors has converted AIS management a concerning major public health problem, requiring an urgent and comprehensive approach. 

Early restoration of cerebral blood flow with EVT, in combination or not with IVT, remains the most effective maneuver for salvaging ischemic brain tissue in patients with AIS. However, not all patients with AIS are eligible for these procedures. Apart from reperfusion techniques, there is still not enough evidence to support the use of other techniques to fight against secondary brain damage and enhance the outcomes of these patients.

In order to address to this demand, examining the underlying mechanisms of AIS is a key. For decades, research has focused on single-target therapies, making it difficult to obtain positive results. As we have previously discussed, stroke is a complex disease that includes a large number of signaling pathways. Thus, searching for pleiotropic agents that are capable of interfering in different targets from different mechanisms within the ischemic cascade, or even different cells from the neurovascular unit, will be very important. 

There are a wide range of emerging novel strategies to treat AIS. FDA approval of these drugs and their introduction in clinical practice will change drastically the future of this group of patients, expanding the therapeutic possibilities to a larger population and enhancing their outcomes. Furthermore, future advances that include cerebroprotective strategies in the clinical practice are expected to considerably prolong the therapeutic time for cerebral penumbra´s reperfusion, leading to an important reduction of the cerebral permanent damage and neurological deficit.

## 8. Limitations of the Work

This review has a few limitations. First, it only focused on studies that were published in English. Second, this manuscript has been written with the scientific knowledge published during the last 15 years in mind.

## Figures and Tables

**Table 1:** Main causes of AIS. * Main sources of cardiac embolism.

Atherosclerotic Plaque	Cardiac Embolism	Others
- Embolism (aortic arch/cervical arteries) - In-situ thrombosis (intracranial arteries)	- Atrial fibrillation * - Myocardial akinesia * - Endocarditis * - Foramen ovale*	- Arterial dissection - Vasculitis - Antiphospholipid syndrome - Essential thrombocytosis - Polycythemia

## Data Availability

Not applicable.
